# Supplementary material for: Cytotoxic Potential of Alternaria tenuissima AUMC14342 Mycoendophyte Extract: A Study Combined with LC-MS/MS Metabolic Profiling and Molecular Docking Simulation
Source: Curr Issues Mol Biol. 2022 Oct 20;44(10):5067–85. doi: 10.3390/cimb44100344 (PMC9600980; doi:10.3390/cimb44100344)
Supplement: Supplementary file 1 [file cimb-44-00344-s001.zip › cimb-1932319-supplementary.pdf]

Supplementary materials

Cytotoxic Potential of Alternaria tenuissima AUMC14342 Mycoendophyte Extract: A Study Combined with LC-MS/MS Metabolic Profiling and Molecular Docking Simulation

Amal A. Al Mousa [1,*], Mohamed E. Abouelela [2,5*], Abdallah M. A. Hassane [3], Fatimah S. Al-Khattaf [1], Ashraf A. Hatamleh [1], Hadeel S. Alabdulhadi [4], Noura D. Dahmash [1] and Nageh F. Abo-Dahab [3]

*Correspondence: aalmosa@ksu.edu.sa (A.A.); Tel.: +966505389419; m_abouelela@azhar.edu.eg (M.A.) Tel.: +201123565130.

Contents

Figure	Page
**Figure S1**. Validation of docking protocol by complexed ligand redocking.	3
**Figure S2**. MS^2 mass chromatogram of Cyclo-Ala-Pro-Diketopiperazine.	4
**Figure S3.** MS^2 mass chromatogram of (S)-alternariphent A.	4
**Figure S4.** MS^2 mass chromatogram of Alternapyran.	5
**Figure S5.** MS^2 mass chromatogram of Altenuene (4′-Epialtenuene).	5
**Figure S6**. MS^2 mass chromatogram of Solanapyrone G.	6
**Figure S7**. MS^2 mass chromatogram of 4-methoxy-6-methyl-5-(3-oxobutyl)-2H-pyran-2-one.	6
**Figure S8.** MS^2 mass chromatogram of Nacetyltyramine.	7
**Figure S9**. MS^2 mass chromatogram of Versimide.	7
**Figure S10.** MS^2 mass chromatogram of 7-dehydrobrefeldin A.	8
**Figure S11**. MS^2 mass chromatogram of Alternariol.	8
**Figure S12**. MS^2 mass chromatogram of 5-butyl-4-methoxy-6-methyl-2H-pyran-2-one.	9
**Figure S13**. MS^2 mass chromatogram of Altertenuol.	9
**Figure S14.** MS^2 mass chromatogram of 3-O-demethylaltenuisol.	10
**Figure S15**. MS^2 mass chromatogram of (-)-alternarlactam.	10
**Figure S16.** MS^2 mass chromatogram of 2-(N-vinylacetamide)-4-hydroxymethyl-3-ene-butyrolactone.	11
**Figure S17**. MS^2 mass chromatogram of Alternariol-9-methyl ether.	11
**Figure S18**. MS^2 mass chromatogram of Resveratrodehyde C.	12
**Figure S19.** MS^2 mass chromatogram of Solanapyrone P.	12
**Figure S20**. MS^2 mass chromatogram of Atransfusarin.	13
**Figure S21.** MS^2 mass chromatogram of Alternatain A.	13

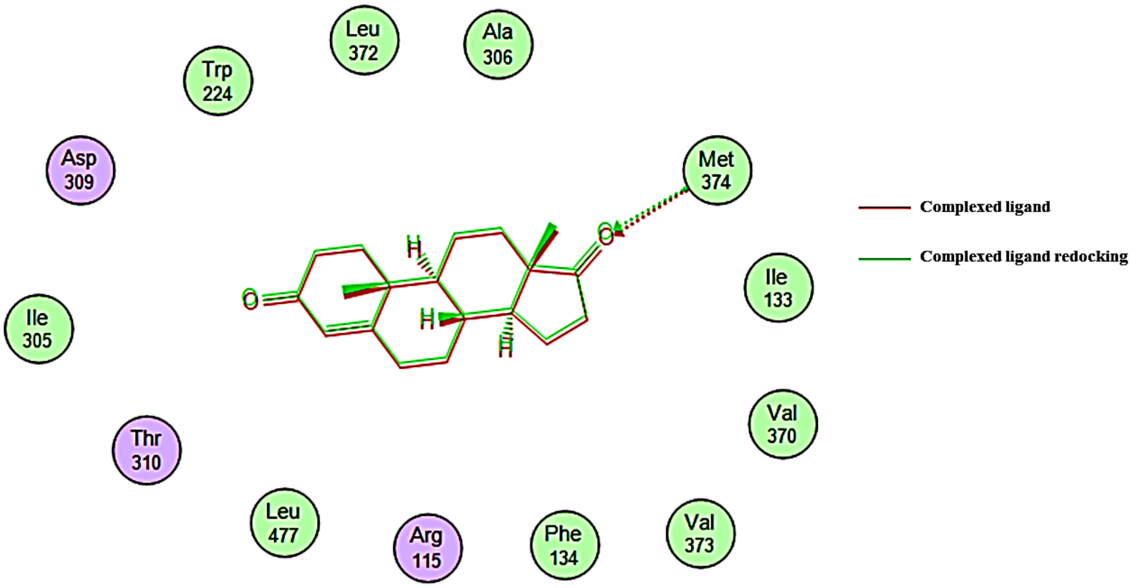

Figure S1. Validation of docking protocol by complexed ligand redocking.

.

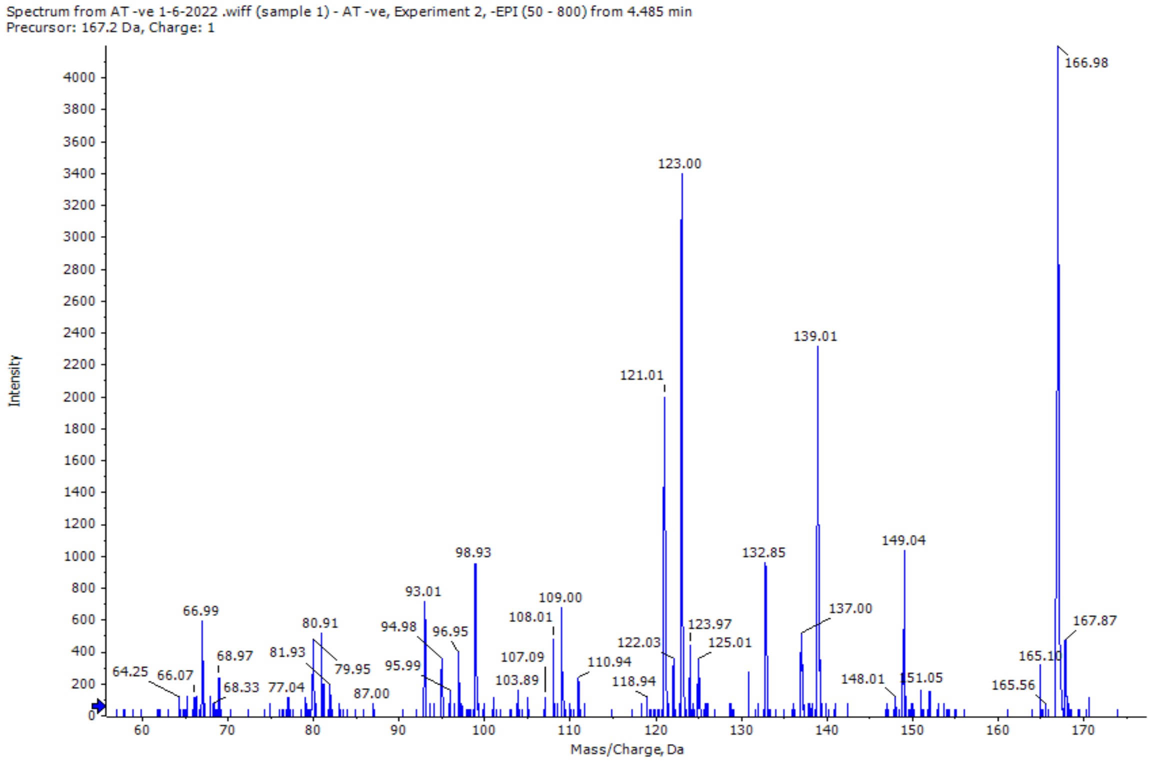

Figure S2. MS^2 mass chromatogram of Cyclo-Ala-Pro-Diketopiperazine.

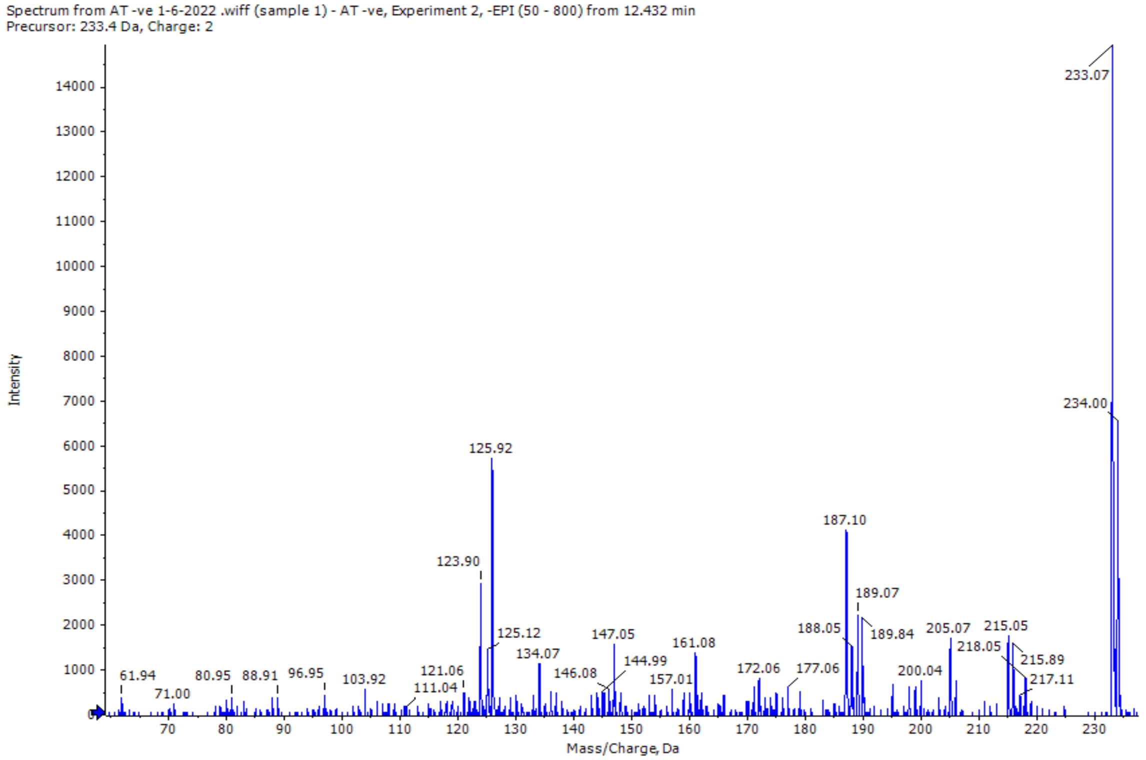

Figure S3. MS^2 mass chromatogram of (S)-alternariphent A.

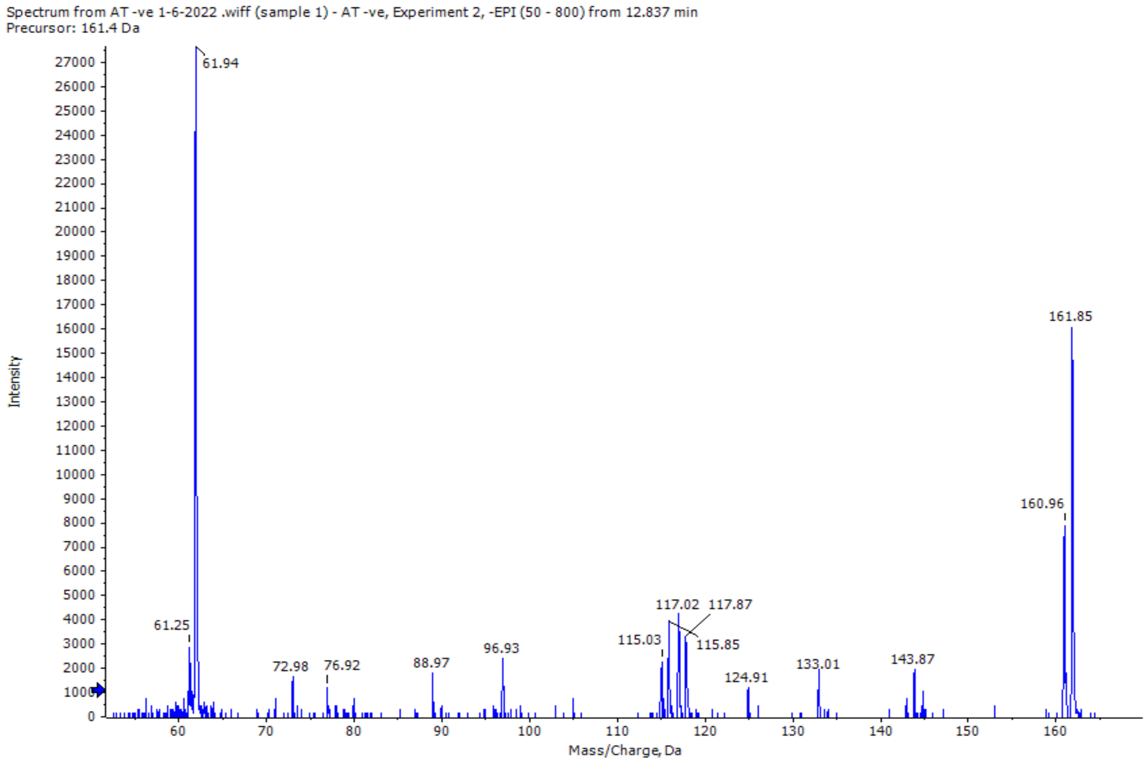

Figure S4. MS[2] mass chromatogram of Alternapyran.

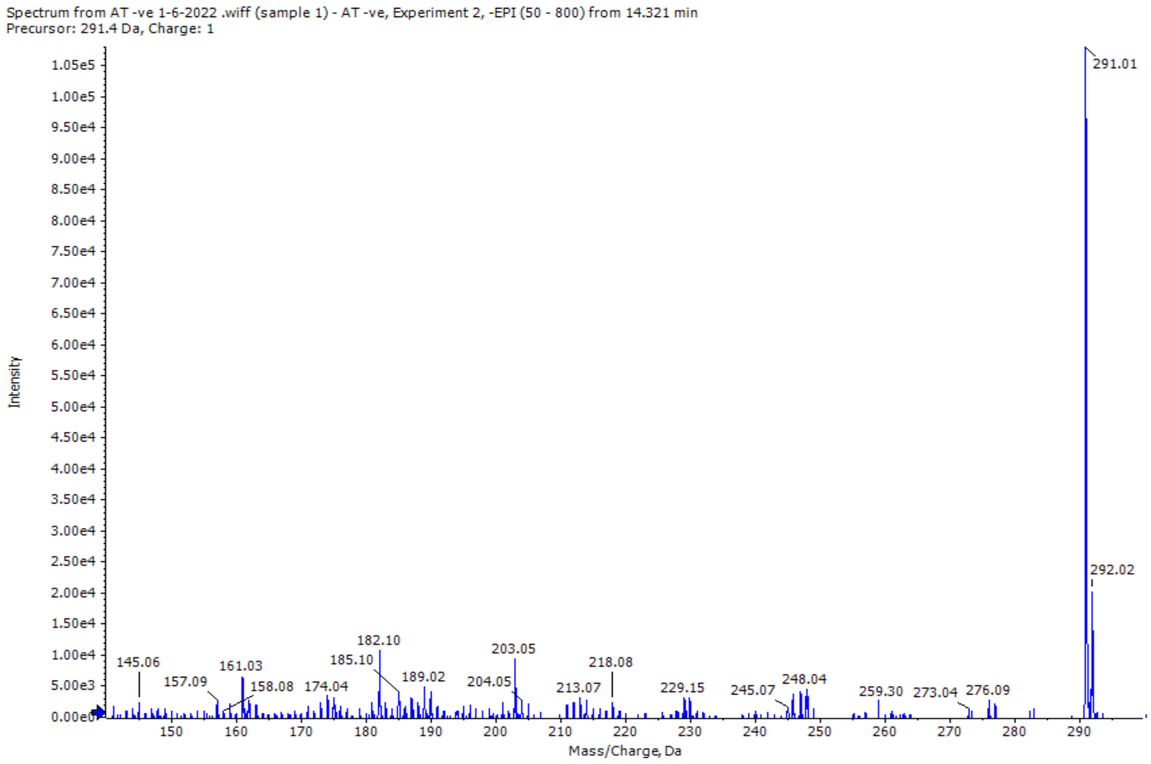

Figure S5. MS[2] mass chromatogram of Altenuene (4′-Epialtenuene).

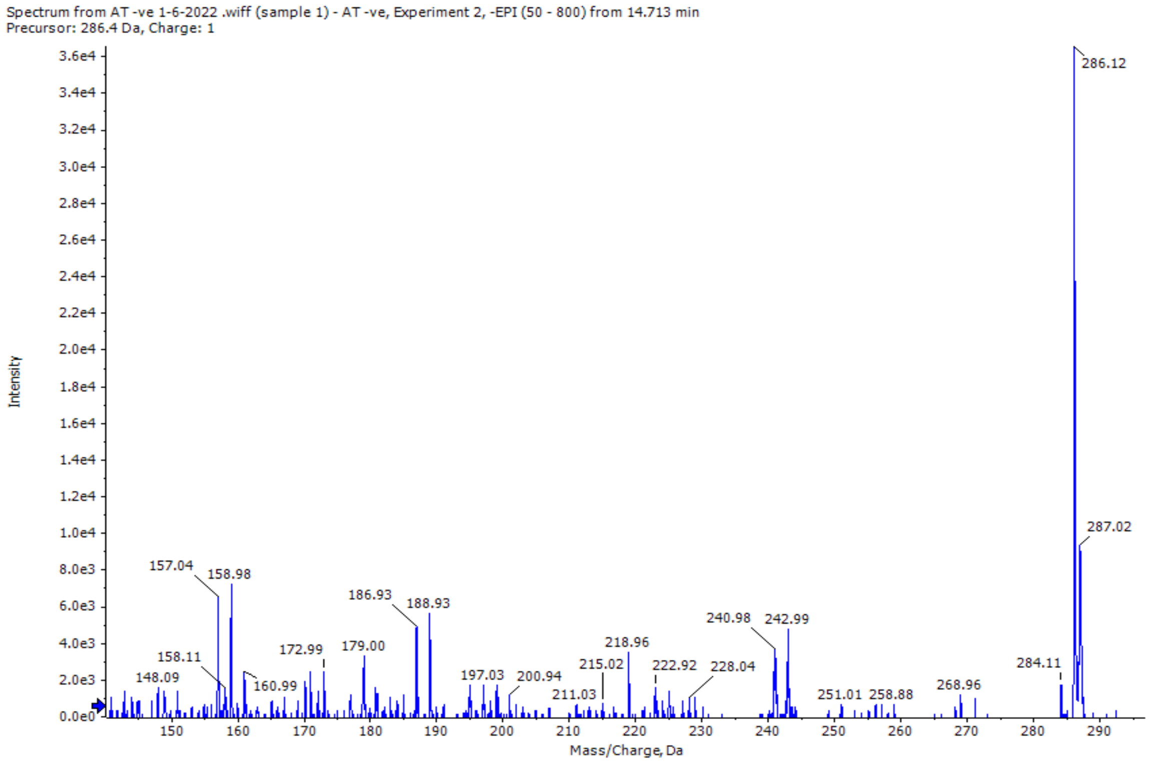

Figure S6. MS² mass chromatogram of Solanapyrone G.

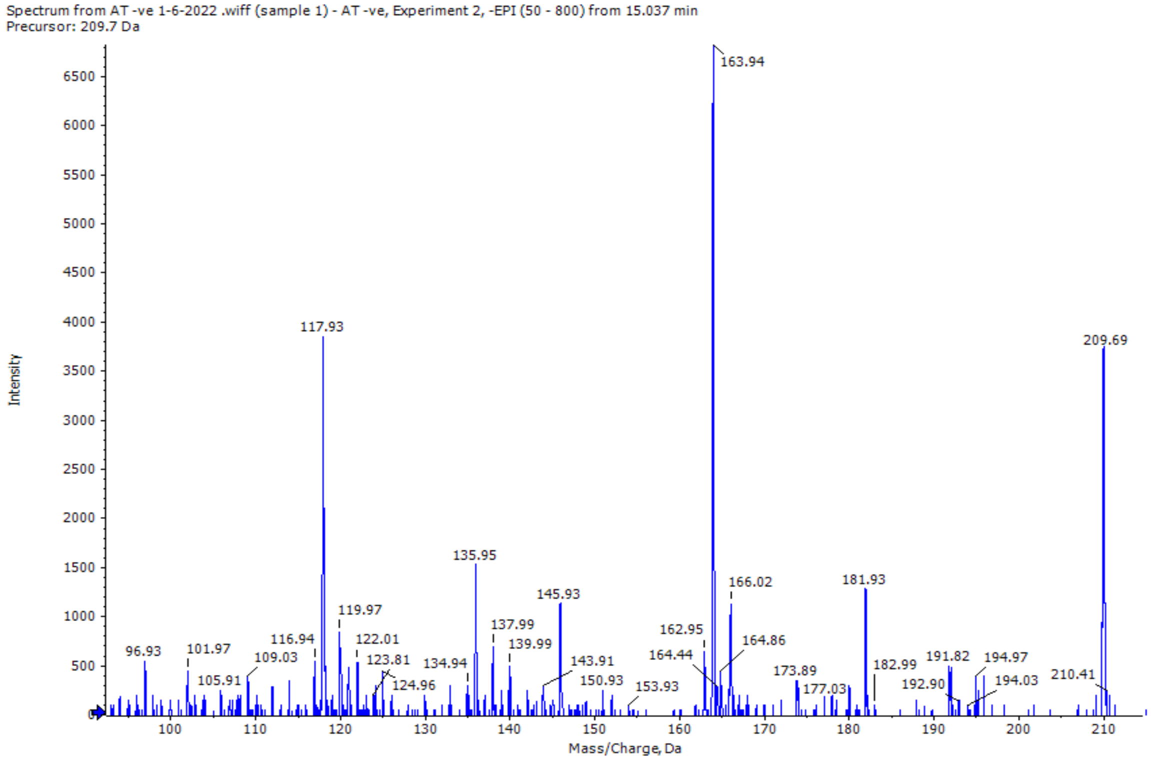

Figure S7. MS² mass chromatogram of 4-methoxy-6-methyl-5-(3-oxobutyl)-2H-pyran-2-one.

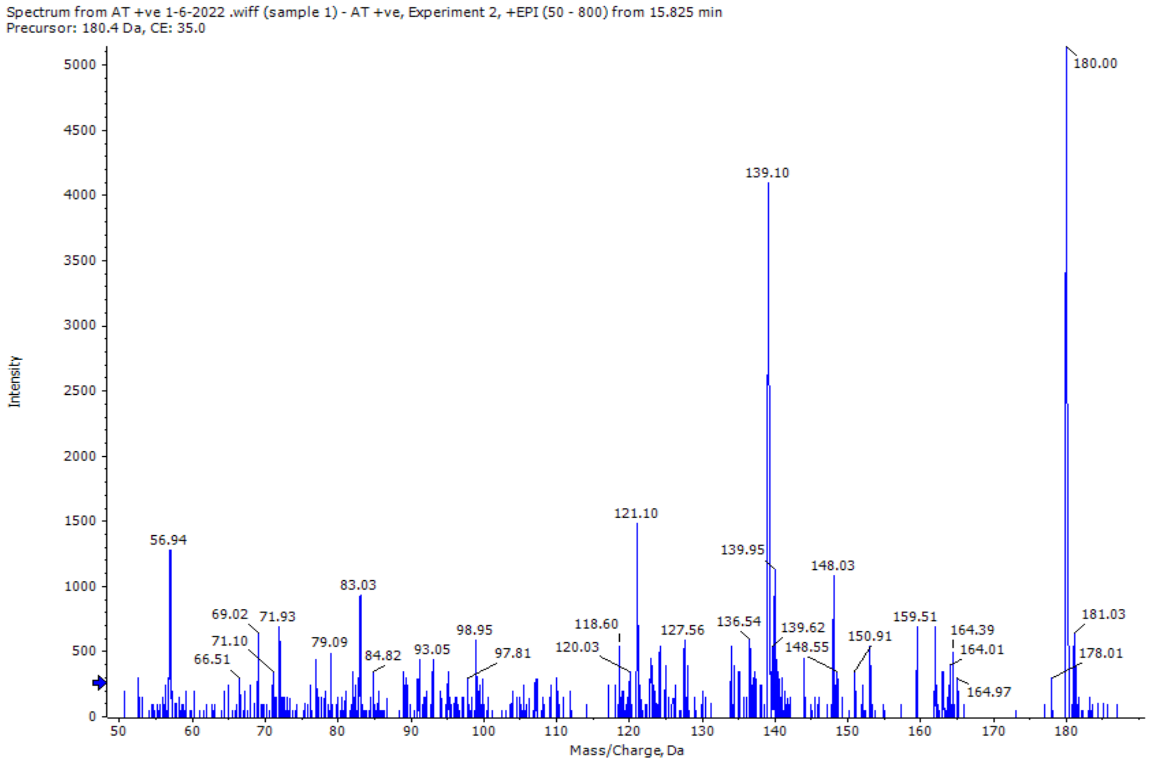

Figure S8. MS^2 mass chromatogram of Nacetyltyramine.

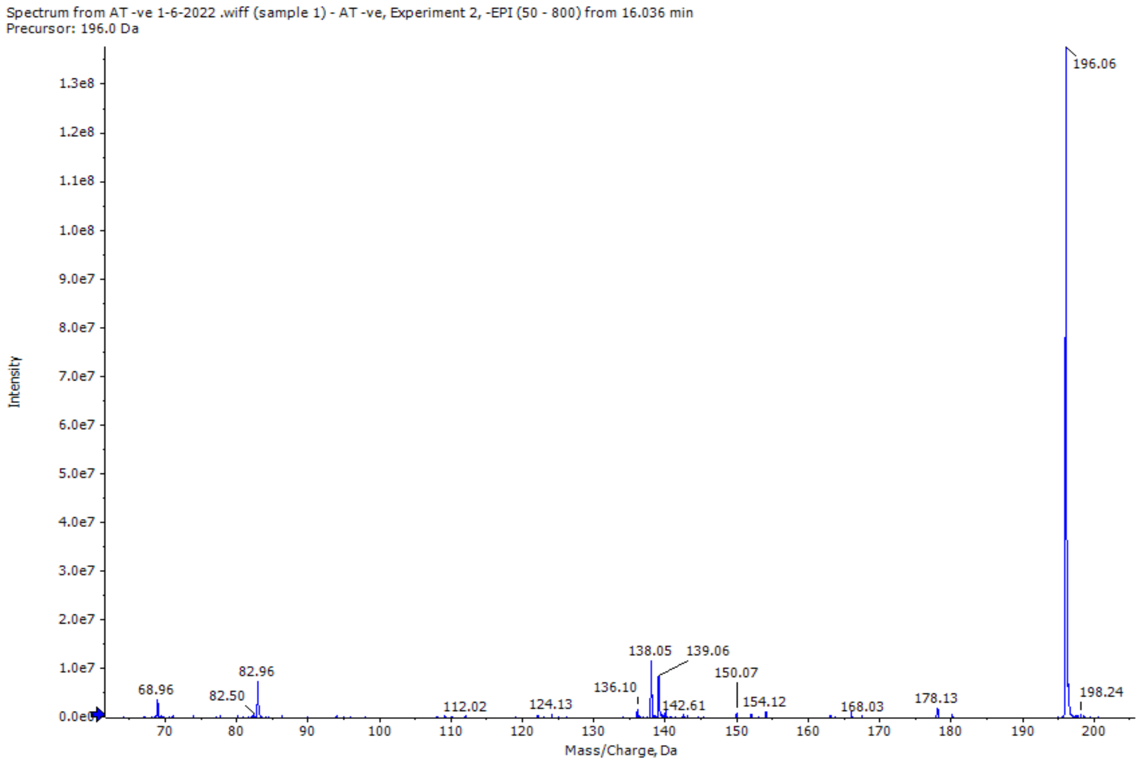

Figure S9. MS^2 mass chromatogram of Versimide.

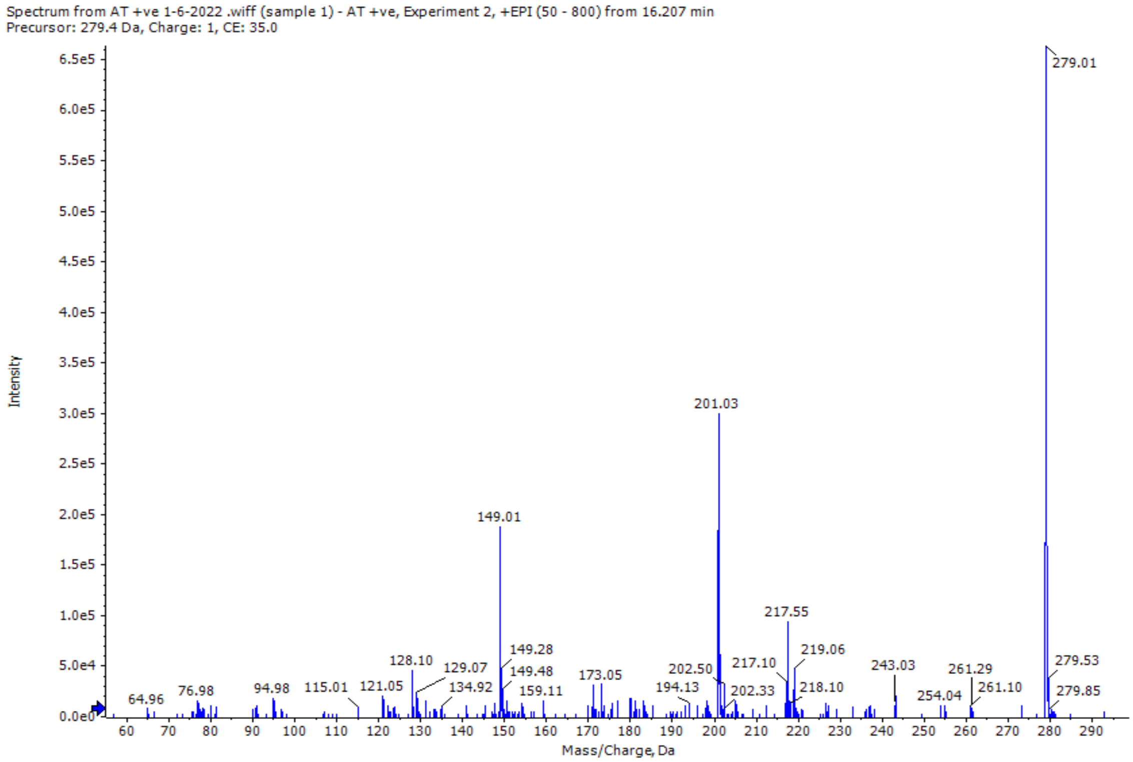

Figure S10. MS² mass chromatogram of 7-dehydrobrefeldin A.

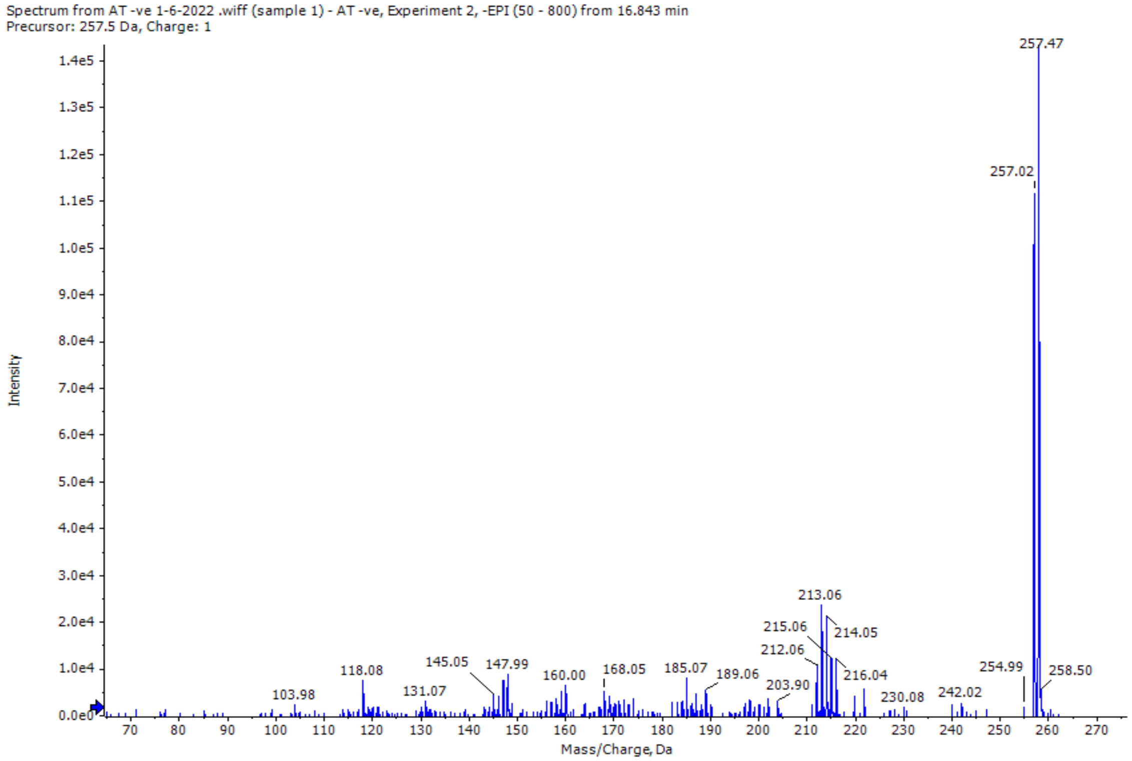

Figure S11. MS² mass chromatogram of Alternariol.

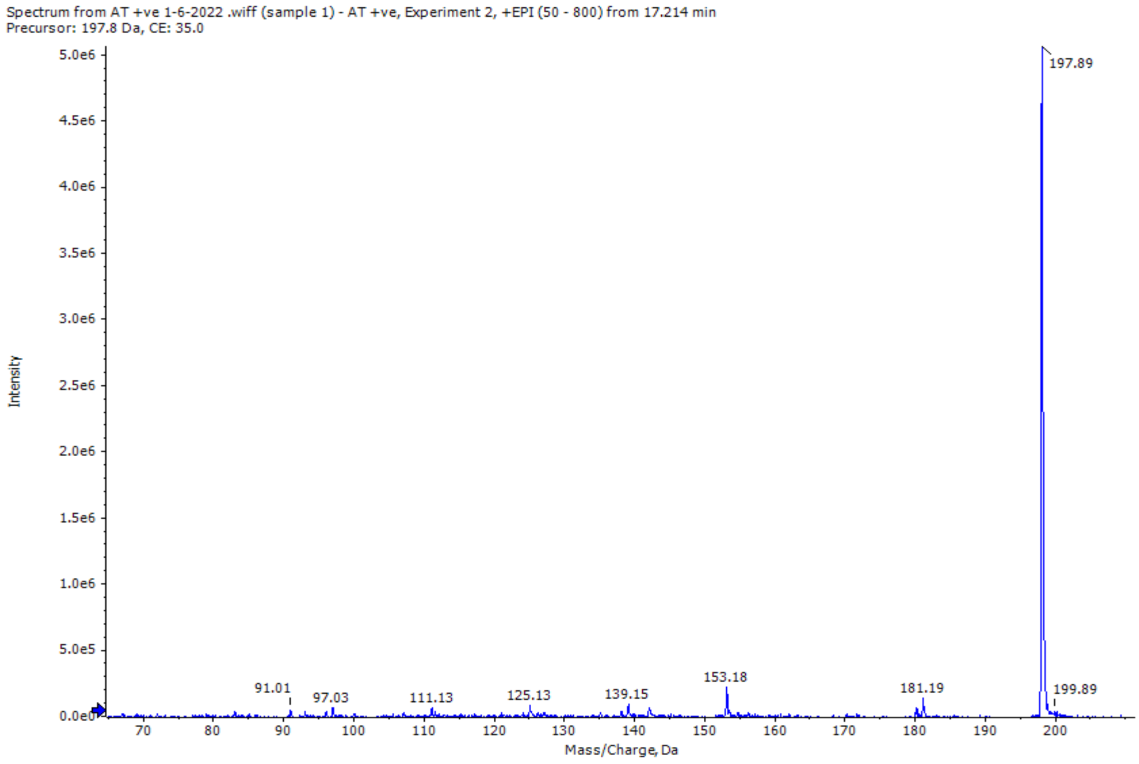

Figure S12. MS² mass chromatogram of 5-butyl-4-methoxy-6-methyl-2H-pyran-2-one.

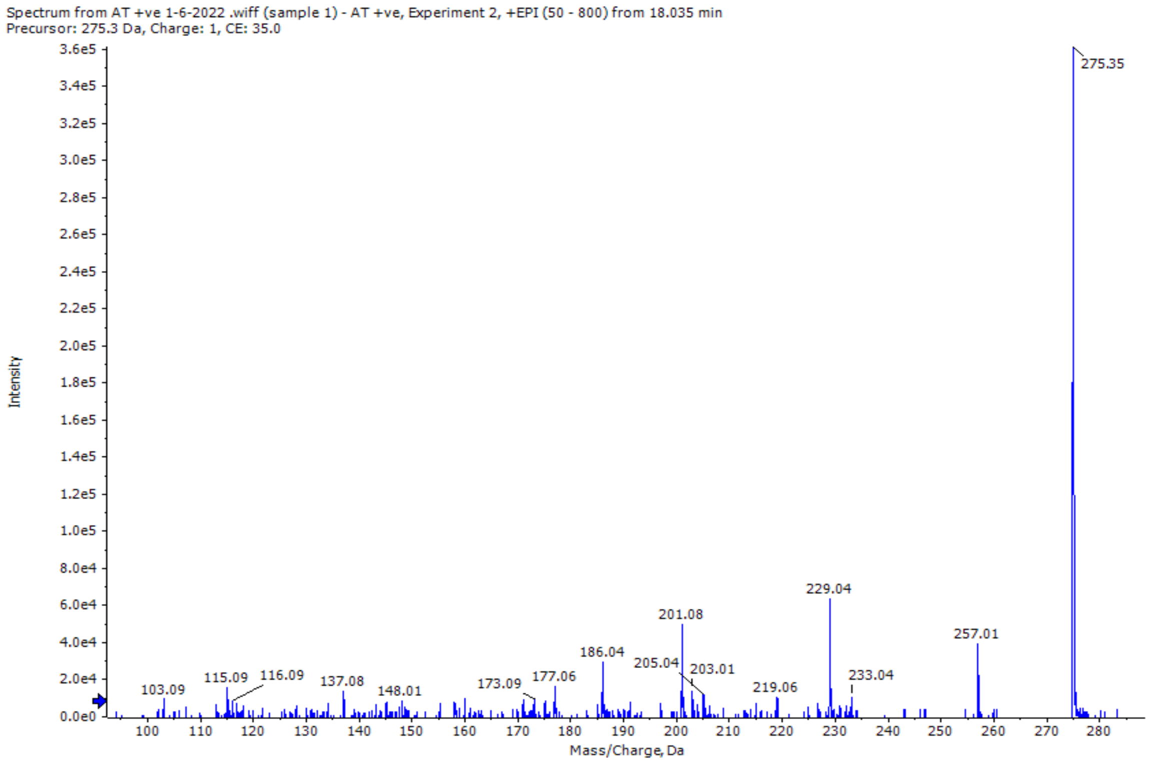

Figure S13. MS² mass chromatogram of Altertenuol.

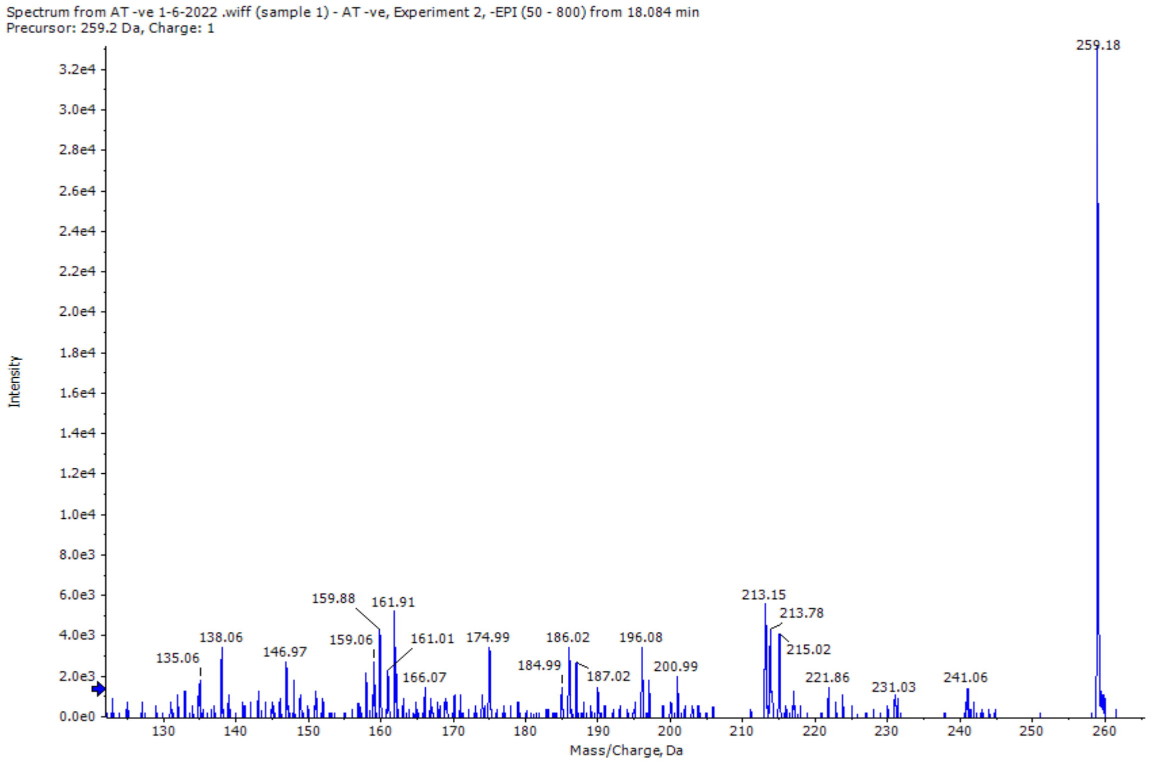

Figure S14. MS[2] mass chromatogram of 3-O-demethylaltenuisol.

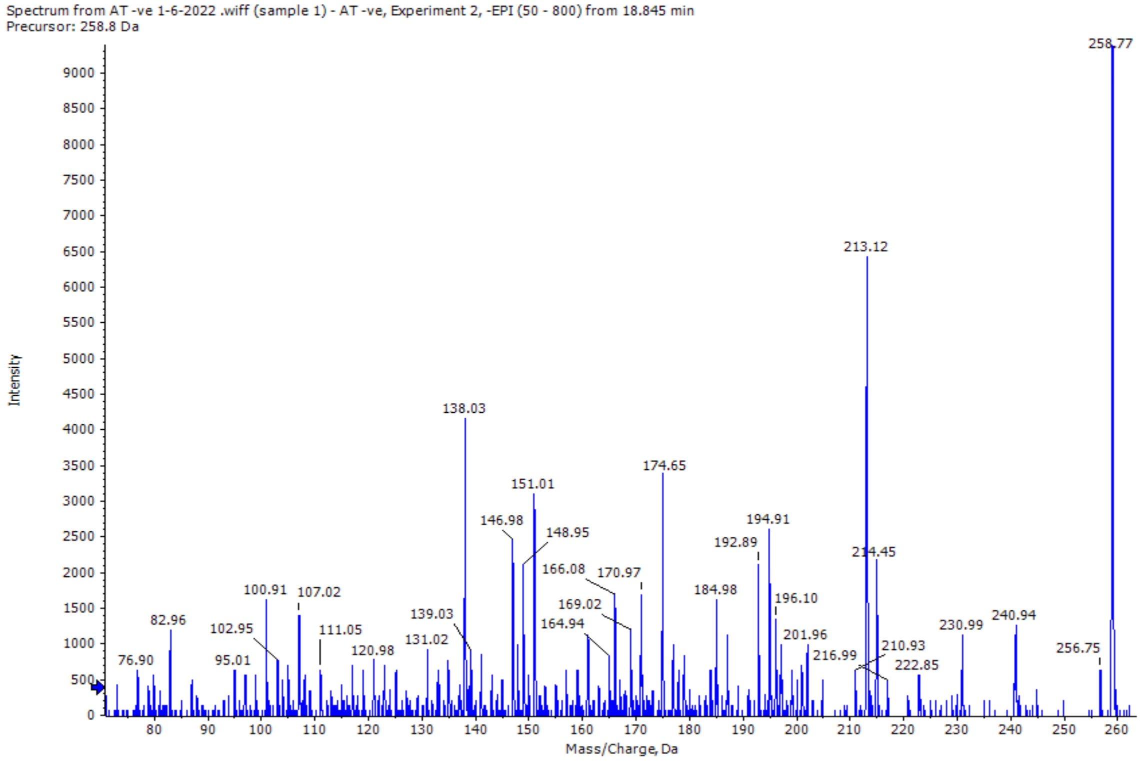

Figure S15. MS[2] mass chromatogram of (-)-alternarlactam.

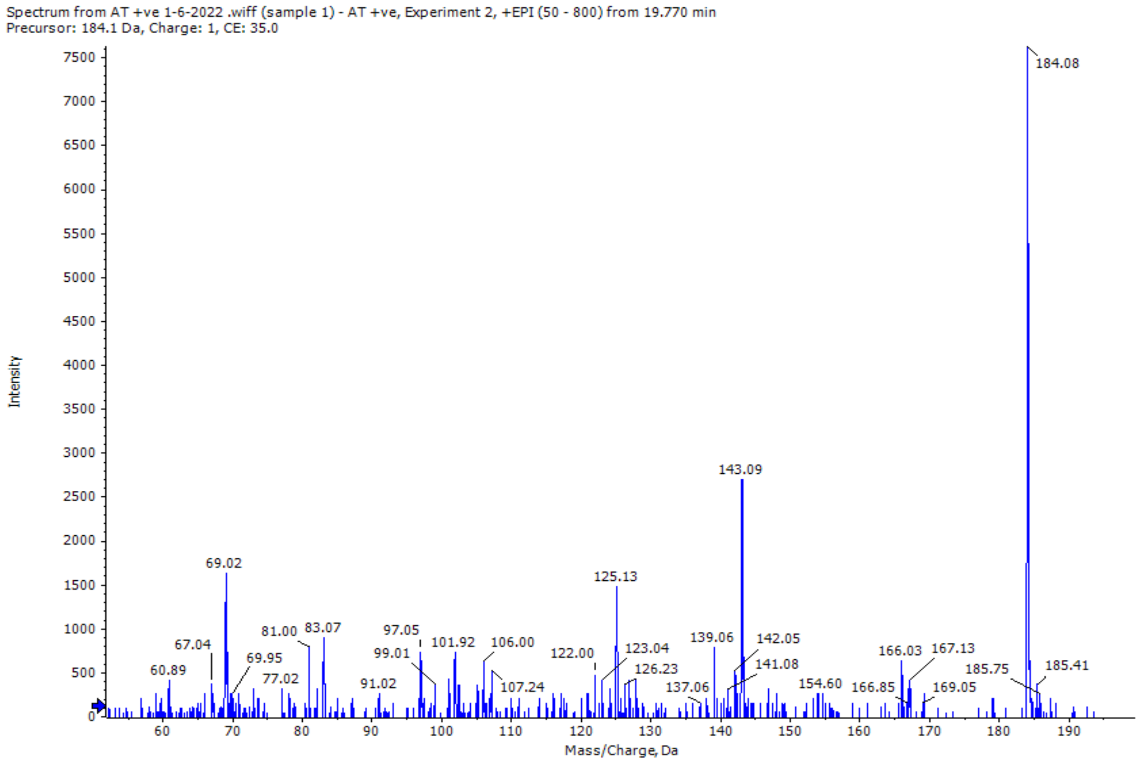

Figure S16. MS² mass chromatogram of 2-(N-vinylacetamide)-4-hydroxymethyl-3-ene-butyrolactone.

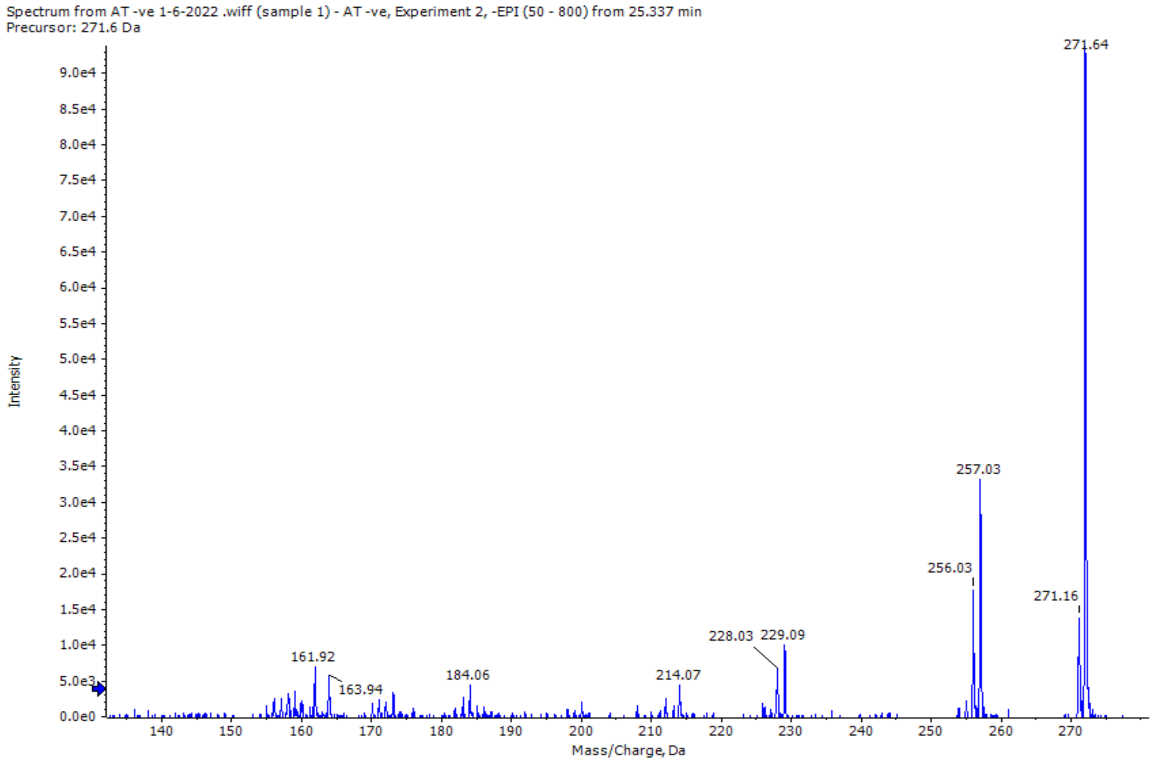

Figure S17. MS² mass chromatogram of Alternariol-9-methyl ether.

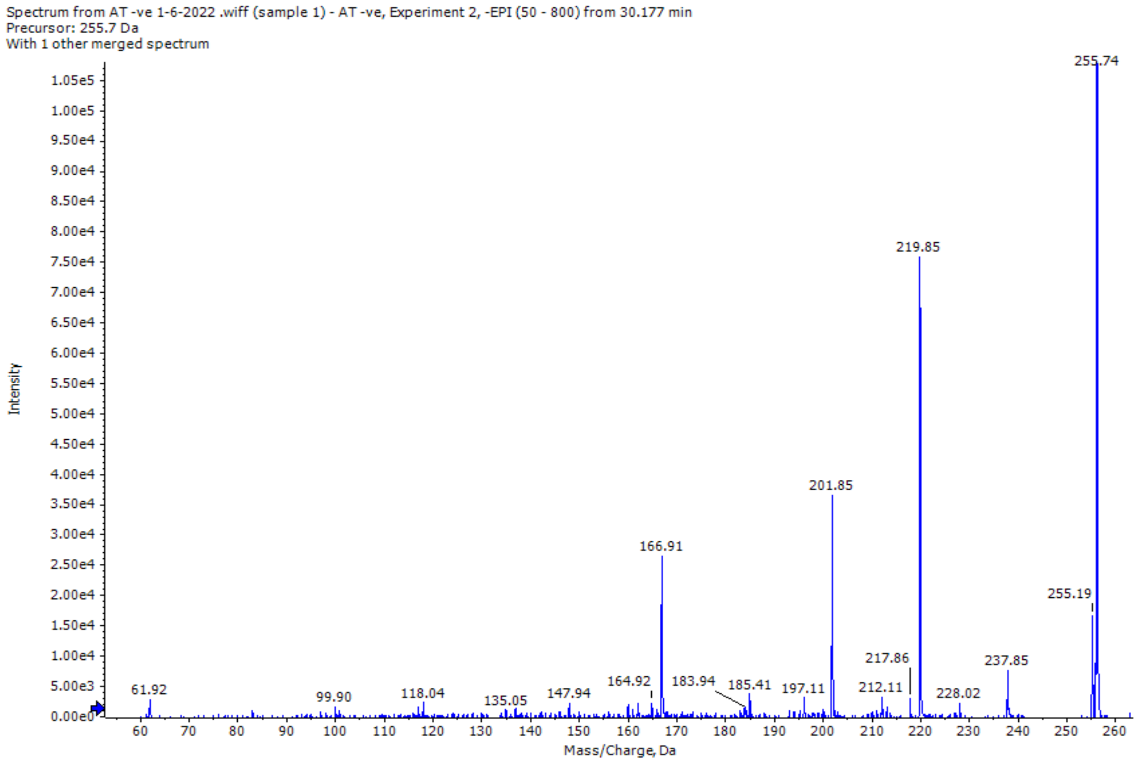

Figure S18. MS² mass chromatogram of Resveratrodehyde C.

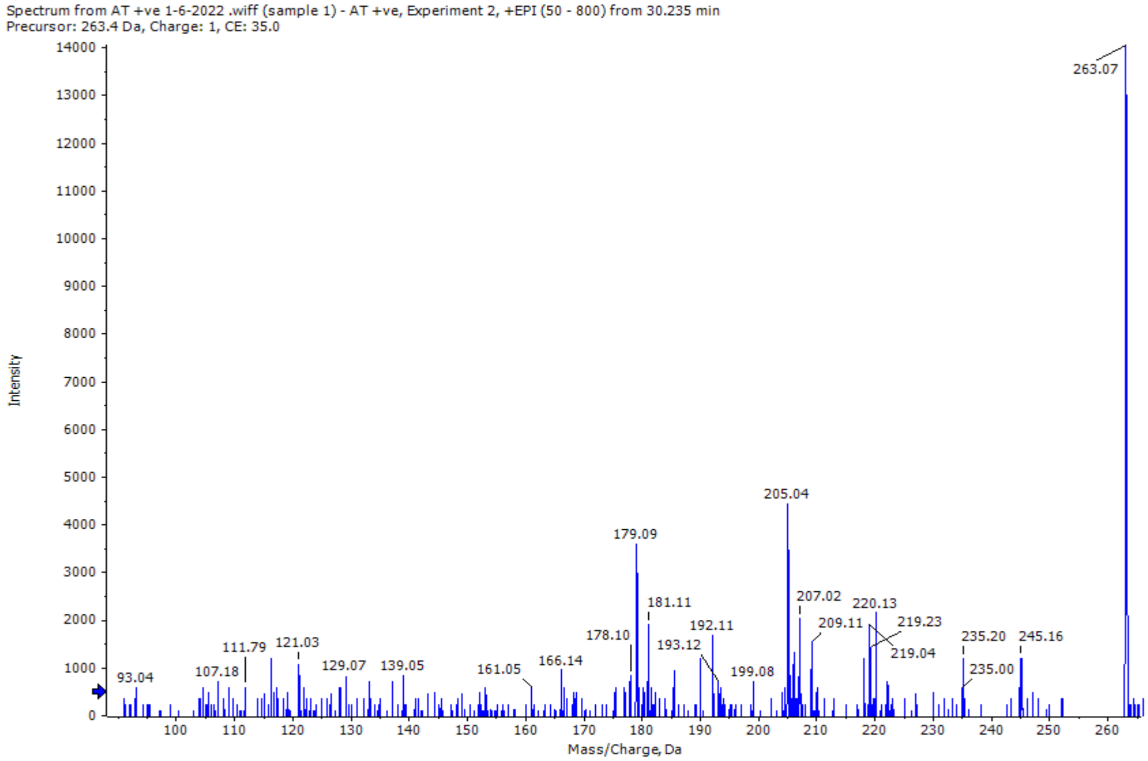

Figure S19. MS² mass chromatogram of Solanapyrone P.

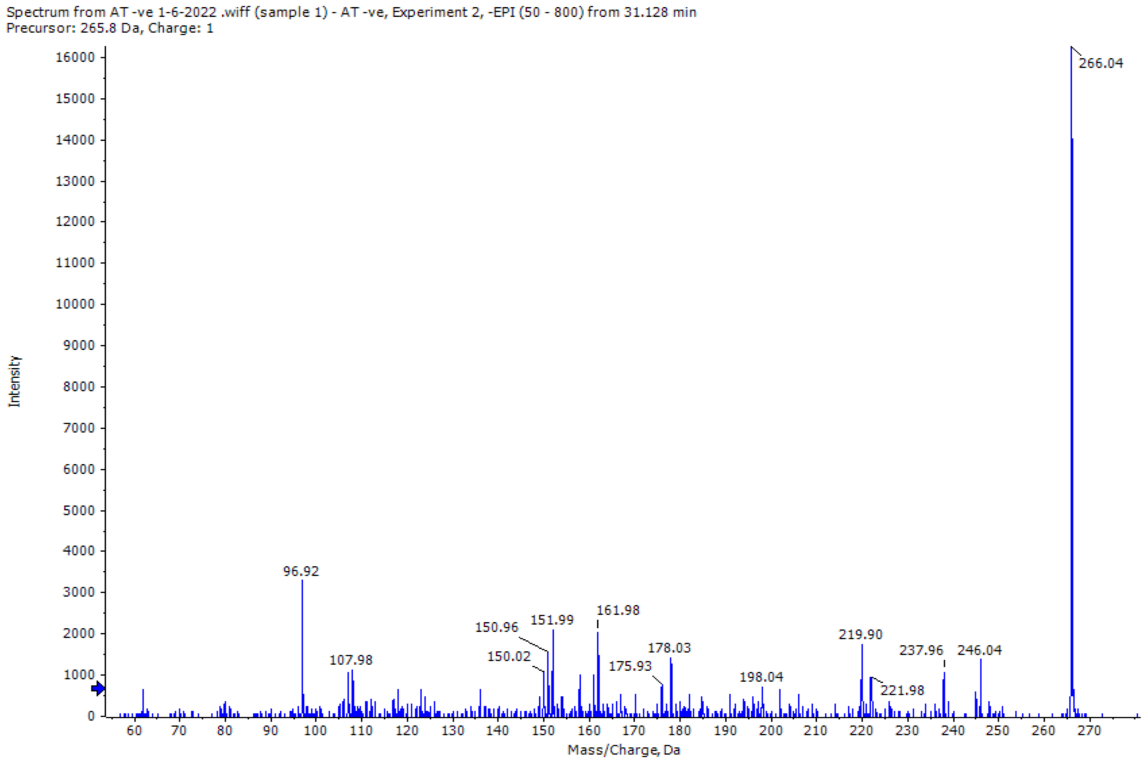

Figure S20. MS² mass chromatogram of Atransfusarin.

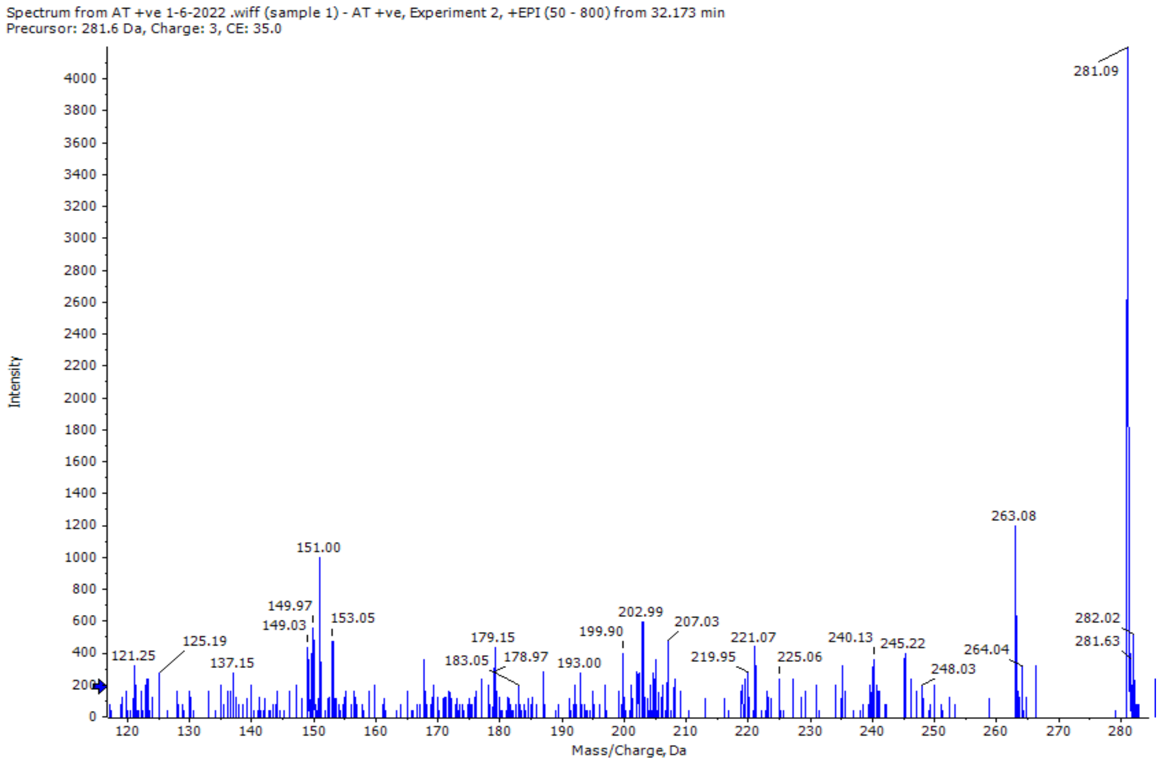

Figure S21. MS² mass chromatogram of Alternatain A.
